# **Heatwave predicts a shady future for insects: impacts of an extreme weather event on a chalk grassland in Bedfordshire, UK**

**DOI:** 10.1007/s10841-024-00556-5

**Published:** 2024-02-05

**Authors:** Matthew P. Hayes, Esme Ashe-Jepson, Gwen E. Hitchcock, Ryan Clark, Josh Hellon, Richard I. Knock, Andrew J. Bladon, Edgar C. Turner

**Affiliations:** 1 Department of Zoology, University of Cambridge, Downing Street, Cambridge, Cambridgeshire, CB2 3EJ UK; 2 Wildlife Trust for Bedfordshire, Cambridgeshire & Northamptonshire, UK

**Keywords:** Climate change, Lepidoptera, Microclimate, Refugia, Topographic manipulation

## Abstract

Climate change is set to become one of the leading causes of biodiversity loss worldwide, with extreme weather events projected to increase in frequency. Ectothermic animals such as insects are at particular risk, especially when they are isolated and unable to move through the landscape to track suitable climate. To protect such taxa, it is important to understand how they are impacted by extreme weather events and whether management could provide effective microclimate refuges. However, potential management interventions remain untested for many species. Here, we show that the extreme high temperatures experienced in the UK on 19th July 2022 resulted in a community of butterflies becoming inactive, but that shaded areas, including artificial slopes created as part of conservation management for climate change, provided a refuge during this period. Our results indicate that future high temperatures could force butterflies to shelter in the shade, potentially being unable to fly, feed or mate during these periods, with possible long-term impacts, particularly if multiple consecutive high temperature days are experienced.

**Supplementary Information:**

The online version contains supplementary material available at 10.1007/s10841-024-00556-5.

## Implications for Insect Conservation

Producing artificial slopes and integrating patches of scrub within grassland could create an array of microclimates that allow butterflies and other invertebrates to thermoregulate, providing a refuge during extreme weather events. Our findings highlight the dramatic effect of extreme temperatures on insect communities, as well as simple management solutions that could be implemented widely and relatively easily by conservation managers, to counter some of the negative impacts of rising temperatures and extreme weather events.

**Supplementary Information:**

The online version contains supplementary material available at 10.1007/s10841-024-00556-5.

## Introduction

Global mean temperatures are projected to rise by at least 1.5–2.0 °C by 2100 (IPCC 2021), with extreme weather events, such as the heat wave experienced in the UK on 18th and 19th July 2022, set to become increasingly common (WWF 2020; IPCC 2021; Kendon 2022). Rising temperatures are already having ecological impacts, causing changes in species’ distribution and phenology as well as individual activity levels (Dennis and Shreeve 1991; Parmesan et al. 1999; IPCC 2014; Mason et al. 2015; Konvička et al. 2016; Mills et al. 2017; WWF 2020; Fox et al. 2023). In most cases, impacts are projected to get more severe. Indeed, over the next 50 years, climate change is predicted to become one of the leading causes of biodiversity loss worldwide (Newbold 2018; WWF 2020). This may be particularly stark in areas such as Western Europe and the USA, where habitat loss and fragmentation have left populations of many species isolated on fragments of suitable habitat, unable to respond to climate change by moving through the wider landscape, and therefore vulnerable to extinction (Warren et al. 2001; Fox et al. 2015; Holyoak and Heath 2016; Gallé et al. 2022). To protect such species, we need to understand how they are impacted by extreme weather events, and whether management in situ could mitigate any negative impacts.

Temperature alters the performance of ectotherms in a host of different ways, and this has been the topic of extensive study (Huey and Kingsolver 1989; Kingsolver and Gomulkiewicz 2003; Kingsolver and Huey 2008; Angilletta 2009). In particular, small ectothermic animals, such as insects, may be vulnerable to changing temperature regimes. For example, being largely unable to generate their own body heat, insects generally rely on heat from their environment to regulate their metabolism, warm their muscles to optimal temperatures and facilitate movement (Clench 1966; Huey and Kingsolver 1989; Kemp and Krockenberger 2004). Furthermore, the large surface area to volume ratio of insects reduces their ability to retain heat, meaning insect behaviours are often closely tied to the temperature of their immediate environment (Clench 1966; Kingsolver and Watt 1983; Kemp and Krockenberger 2004; Hill et al. 2021). Therefore, changing regional temperatures could have a large impact on insect populations, by altering the amount of time available for insects to be active. Energetically demanding behaviours such as flight (Shreeve 1984; Berwaerts and Van Dyck 2004; Merckx and Van Dyck 2005; Tsubaki and Samejima 2016; Hayes et al. 2019; Kral-O’Brien et al. 2021) are likely to be most affected, and links with feeding, mate acquisition and oviposition mean flight performance has the potential to be closely tied to population-level fitness (Huey and Kingsolver 1989; Berwaerts and Van Dyck 2004; Sunday et al. 2014; Woods et al. 2015; Evans et al. 2019; Hill et al. 2021). As well as indirectly impacting fitness through altered activity levels, there is also evidence that extreme temperatures can directly impact fitness, through sub-lethal effects such as reduced egg viability and sterility (Rohde et al. 2017; van Heerwaarden and Sgrò 2021), and can eventually lead to the death of individuals (Harvey et al. 2023). Prolonged changes in temperature, where extremes are experienced over multiple successive days, might be expected to have larger impacts on fitness than shorter-term changes, but even short-term extreme weather events can impact invertebrate communities (Nakonieczny et al. 2007; Thakur et al. 2022; Harvey et al. 2023).

Many butterfly species are acutely sensitive to environmental change, having short life spans and complex life cycles, with each life stage having distinct habitat requirements (Fleishman et al. 2005; Thomas 2005). Different species of butterfly react differently to rising air temperatures, with behaviour being affected in different ways (Angilletta 2009; Kleckova and Klecka 2016; Bladon et al. 2020). However, general patterns can be observed, whereby activity levels increase as temperatures warm, followed by activity levels peaking and eventually falling as conditions become too hot (Clench 1966; Kingsolver and Watt 1983; Kemp and Krockenberger 2004; Angilletta 2009; Kleckova and Klecka 2016; Evans et al. 2019). The UK is at the northern range limit for many species of butterfly and, as such, we may expect an initial increase in activity with rising temperatures (Fox et al. 2023). However, evidence suggests that species in the UK, such as the meadow brown (*Maniola jurtina*), may already be approaching their thermal optimum (Evans et al. 2019). Butterflies and moths can respond to temperature changes using a combination of physiological, morphological and behavioural adaptations (Clench 1966; Kingsolver and Watt 1983; Kemp and Krockenberger 2004; Angilletta 2009; Hill et al. 2021). For example, shivering is widespread amongst moths but also observed in some butterfly species. In cold conditions, individuals use rapid vibrations of their wings and thoracic flight muscles to generate metabolic heat (Clench 1966; Srygley 1994). Another commonly observed adaptation is individual butterflies changing the orientation of their wings towards or away from the sun, to increase or reduce interception of radiation (Clench 1966). Coupling this behaviour with adaptations of shape and colouration can make butterfly wings, particularly the base of wings close to the thorax, powerful tools for thermoregulation (Watt 1968; Wasserthal 1975; Kingsolver and Watt 1983; Kemp and Krockenberger 2004; Angilletta 2009; Shanks et al. 2015; Hill et al. 2021). In these ways, butterflies are able to alter their body temperature from that of their surroundings. Nonetheless, being restricted in their ability to generate their own body heat or to use physiological cooling mechanisms means that butterflies and other insects are likely to be strongly impacted by environmental temperatures. This is supported by studies that have reported local population extinctions in butterflies that are linked to global warming and weather anomalies (Nakonieczny et al. 2007).

Many insects also make use of variable microclimates in their local area to thermoregulate (Angilletta 2009; Sunday et al. 2014; Woods et al. 2015; Hill et al. 2021). Heterogenous habitats are needed to protect communities of butterflies and provide the diverse range of resources required by different species, sexes and life stages (Futuyma 1976; Shreeve 1984; Shreeve 1986; McNeely and Singer 2001; Tews et al. 2004; Dennis 2010; Slamova et al. 2013). Indeed, much successful butterfly conservation to date has focussed on providing for these diverse habitat requirements (Dennis 2010). However, in the face of climate change, a diverse thermal environment may represent an equally important resource (Sunday et al. 2014; Woods et al. 2015; Sears et al. 2016; Suggitt et al. 2018). Temperature and humidity regimes can vary widely across landscapes, with areas experiencing different microclimates depending on slope, aspect, vegetation height and structure, which impact levels of shade and shelter (Shreeve 1984; Angilletta 2009; Oliver 2014; Sunday et al. 2014; Suggitt et al. 2015; Woods et al. 2015; Kleckova and Klecka 2016; Sears et al. 2016; Bramer et al. 2018; Suggitt et al. 2018; Pincebourde and Woods 2020). These microclimates can be used by insects to warm up and cool down. For example, if ambient conditions are too warm, butterflies can seek cooler shaded areas beneath vegetation to cool down (Clench 1966; Kingsolver and Watt 1983; Kemp and Krockenberger 2004; Kleckova and Klecka 2016; Hill et al. 2021). This means that butterfly habitat use and activity levels are strongly tied to microclimatic variables, which can vary on a small scale (Shreeve 1984; Berwaerts and Van Dyck 2004; Angilletta 2009; Kleckova and Klecka 2016). If species occupy habitats that provide a range of different microclimates, they may be buffered against the negative impacts of temperature change (Suggitt et al. 2012; Greenwood et al. 2016; Bladon et al. 2020). Therefore, enhancing vegetation and topographic heterogeneity in situ, through altered vegetation management practices or the creation of artificial topography (sometimes called “butterfly banks” (Butterfly Conservation 2022), to increase the range of microclimates available, could prove a key tool for protecting species into the future (Oliver 2014; Suggitt et al. 2015; Kleckova and Klecka 2016; WTBCN 2021).

Despite many studies investigating butterfly responses to temperature change (Clench 1966; Kingsolver and Watt 1983; Shreeve 1984; Kemp and Krockenberger 2004; Kleckova and Klecka 2016) and suggesting that the maintenance of suitable microclimates will be important for supporting adaptation to climate change (Sunday et al. 2014; Woods et al. 2015; Kleckova and Klecka 2016; Sears et al. 2016; Suggitt et al. 2018), no studies have yet tested experimentally whether artificial fine-scale habitat manipulation can successfully create microclimatic refugia for Lepidoptera (Bladon et al. 2023). In addition, more data from extreme weather events are needed to explore the effect of extreme high temperatures on butterflies in a natural setting. In this paper, we use a case-study approach to assess how predicted future temperatures and extreme weather events could impact activity levels, local habitat choice, and temperature control ability of an entire butterfly community. We used the extreme heatwave experienced in the UK on 19th July 2022, when national temperatures reached a new record of 40.3 °C, as a unique snapshot of possible future regional temperatures under projected climate change (Kendon 2022). This unusual opportunity allowed us to assess: (a) the effect of extreme temperatures on butterfly behaviour in the field, and (b) the effectiveness of two potential management solutions for protecting butterflies from heat waves (interspersing homogeneous, flat grassland with scrub and creating experimental butterfly banks with a diverse topography). We addressed the following related questions:

1. Does the probability of butterflies displaying behaviours involving flight decrease at extreme high air temperatures?
2. Does the probability of butterflies using shade increase at extreme high air temperatures?
3. Does the probability of butterflies being found within 5 m of shelter increase at extreme high air temperatures?
4. Does the difference between butterfly body temperature and air temperature reduce at extreme high air temperatures?
5. Do butterflies select relatively cooler microclimate temperatures at extreme high air temperatures?

## Methods

### Study site and experimental manipulation

The study was conducted at Pegsdon Hills and Hoo Bit nature reserve, in Bedfordshire, UK (− 0.37020, 51.95354), a chalk grassland site owned and managed by the Wildlife Trust for Bedfordshire, Cambridgeshire and Northamptonshire (WTBCN 2023). In September 2021, four experimental butterfly banks were built on the reserve, as part of the Banking on Butterflies Project (WTBCN 2021). Chalk earth scrapings were built in situ into 1.5-m high banks in the shape of capital letter E’s (Supplementary data Fig. 1A). The banks are approximately 16-m long and 7-m wide, and each include a 16 × 5-m adjacent area of cleared vegetation with no topographic manipulation (Supplementary data Fig. 1B). Each bank faces the direction of a different cardinal compass point. Vegetation has been allowed to recolonise the banks naturally. Before the banks were built, this area of the reserve was homogenous ex-arable grassland, sloping by approximately 10 degrees to the east (Supplementary data Fig. 1C). The banks are part of an ongoing project investigating the impact of artificially altering topography, testing whether this form of management can be used to alter microclimates and how this, in turn, effects the community composition of plants and insects.

### Data collection

Data were collected from the field containing the butterfly banks (Supplementary data Fig. 1Ci) and adjacent scrub (Supplementary data Fig. 1Cii). The site was surveyed for adult butterflies and day-flying moths on six days over a 15-day period from 19th July – 3rd August 2022, with a morning and afternoon survey each day. The surveys on 19th July were conducted to specifically assess how butterflies were affected by the hottest day on record in the UK. The five days of subsequent surveys gathered data under lower, more normal, temperatures for comparison. On all five of the ‘normal’ cooler days, recorded air temperature ranges overlapped, whereas every temperature recorded on 19th of July was above this range (Supplementary data Fig. 2). Morning surveys were conducted between 08:30 and 11:20, an hour to an hour and a half earlier than butterfly surveys usually start (Butterfly Conservation 2023), while afternoon surveys were conducted between 12:50 and 15:30. While butterflies are active outside of these time windows, recording at these two discrete times of day gave us representative observations to assess the effect of the heat wave on the timing of butterfly activity.

During each survey, the entire study area was systematically searched for all butterfly species (and day-flying moths), except during the first morning, on 19th July, when recordings were not taken near a large section of scrub that provides shade on the eastern edge of the site (Supplementary data Fig. 1Cii). However, we noticed a large amount of butterfly activity in the edge of the scrub after completing the first survey, and therefore included this in all later surveys. During surveys, the area of each shade patch encountered was estimated by eye, to account for the size and location of these changing with the position of the sun. The area of all patches of shade was summed to give a total estimate of available shade within the survey area during each survey.

Recorders walked up and down the study site in parallel lines, getting within 10 m of all locations, and gently waving a butterfly net to flush out any butterflies not initially seen. The starting point for each survey was varied to ensure recorders were not at the same location at the same time each survey. Any butterfly seen within 10 m was recorded and, if possible, captured using a butterfly net. For each butterfly, we recorded the species, whether it was flying when first spotted (a measure of activity level), whether or not it was located in shade, and whether or not it was within 5 m of a butterfly bank (the extent around the banks that were manipulated when they were created) or scrub, both of which provided shelter. If the butterfly was caught, we immediately recorded its body temperature by touching a fine (0.25 mm) mineral-insulated type K thermocouple with hand-held indicator (Tecpel Thermometer 305B) to its thorax, as well as recording air temperature at waist height in the shade at the same location. If the captured butterfly was first observed perching on a substrate, the temperature of both the substrate (measured by placing the end of the thermocouple on the substrate) and its immediate microclimate (measured by placing the end of the thermocouple 1 cm above the substrate) were also recorded (following Bladon et al. (2020).

### Statistical analyses

All statistical analyses were performed using R version 4.2.2. (R Core Team 2022) within R Studio version 2022.07.2 Build 576 (R Studio Team 2022). The additional packages used were ‘dplyr’ (Wickham et al. 2022a), ‘car’ (Fox et al. 2022), ‘ggplot2’ (Wickham et al. 2022b), ‘cowplot’ (Wilke et al. 2020) and ‘RColorBrewer’ (Neuwirth et al. 2022). Maps were produced using QGIS (QGIS Development Team 2022).

#### Questions 1–3: does the probability of butterflies displaying behaviours involving flight decrease at extreme high air temperatures? Does the probability of butterflies using shade increase at extreme high air temperatures? Does the probability of butterflies being found within 5 m of shelter increase at extreme high air temperatures?

To investigate how rising air temperature affected butterfly flight activity, use of shade and shelter, three separate logistic regressions were fitted. **(1)** Flight activity (flying or not when first observed), was used as the response variable, and air temperature at the time of recording as the explanatory variable. Air temperature was included as a quadratic term to account for the fact that activity is likely to increase with air temperature at low temperatures, but decrease again at high air temperatures (Clench 1966; Kingsolver and Watt 1983; Kemp and Krockenberger 2004; Angilletta 2009; Kleckova and Klecka 2016; Evans et al. 2019). **(2)** Location of each butterfly (in the shade or not) was used as the response variable, and air temperature at the time it was recorded, the total area of shade recorded during that survey, and their interaction term, as explanatory variables. **(3)** The location of each butterfly (whether or not it was within 5 m of shelter provided by a butterfly bank or scrub) was used as the response variable, and the air temperature at the time of recording as the explanatory variable.

#### Question 4: does the difference between butterfly body temperature and air temperature reduce at extreme high air temperatures?

We fitted a linear regression with butterfly body temperature as the response variable, and air temperature at the time of recording, survey day (a two-level factor: 19th July heatwave or any other cooler day, to ensure morning and afternoon surveys were included in each level) and their interaction term, as explanatory variables. A significant interaction would indicate that the relationship between body temperature and air temperature (the butterflies’ buffering ability, Bladon et al. 2020) was different during the heatwave compared to other days. We first ran this analysis using data from all butterfly species combined, and then repeated it for the two individual species (meadow brown *Maniola jurtina* and common blue *Polyommatus icarus*) with more than 30 records on both 19th July, during the heatwave, and all subsequent cooler days combined.

#### Question 5: do butterflies select relatively cooler microclimate temperatures at extreme high air temperatures?

To investigate how extreme high air temperatures affected microclimate selection, we examined data from all individual butterflies where microclimate and substrate temperatures were recorded, in addition to body and air temperatures. We fitted a two-way ANOVA with temperature as the response variable, and type of temperature measure (air temperature, microclimate temperature, substrate temperature and butterfly body temperature), survey time (a four-level factor, separating morning and afternoon surveys on 19th July heatwave from those on any other cooler day into the categories: Cold Morning, Cold Afternoon, Hot Morning or Hot Afternoon), and their interaction term, as explanatory variables. Tukey post hoc tests were used to identify which specific groups (combinations of the two categorical variables) showed significant differences from one another.

## Results

In total, 926 individuals and 16 species of butterfly and day-flying moth were recorded over the six survey days. The meadow brown (*Maniola jurtina*) was the most common butterfly in each survey and represented 58% (538/926) of all records. The second most common species over the six days was the common blue (*Polyommatus icarus*), which represented 13% (123/926) of all records (Supplementary data Table 1).

On the hottest day of the year, 19th July 2022, the highest air temperature recorded during surveys was 39.3 °C and the highest butterfly body temperature was 43.2 °C, both in the afternoon. The lowest air and butterfly body temperatures recorded that day were 30.1 and 31.3 °C respectively, both in the morning. Across all other survey days, the highest air temperature was 27.9 °C and the highest butterfly body temperature was 35.2 °C. The lowest air and butterfly body temperatures were 17.5 and 19 °C, respectively (Supplementary data Fig. 2).

### Question 1: does the probability of butterflies displaying behaviours involving flight decrease at extreme high air temperatures?

**Fig. 1:**
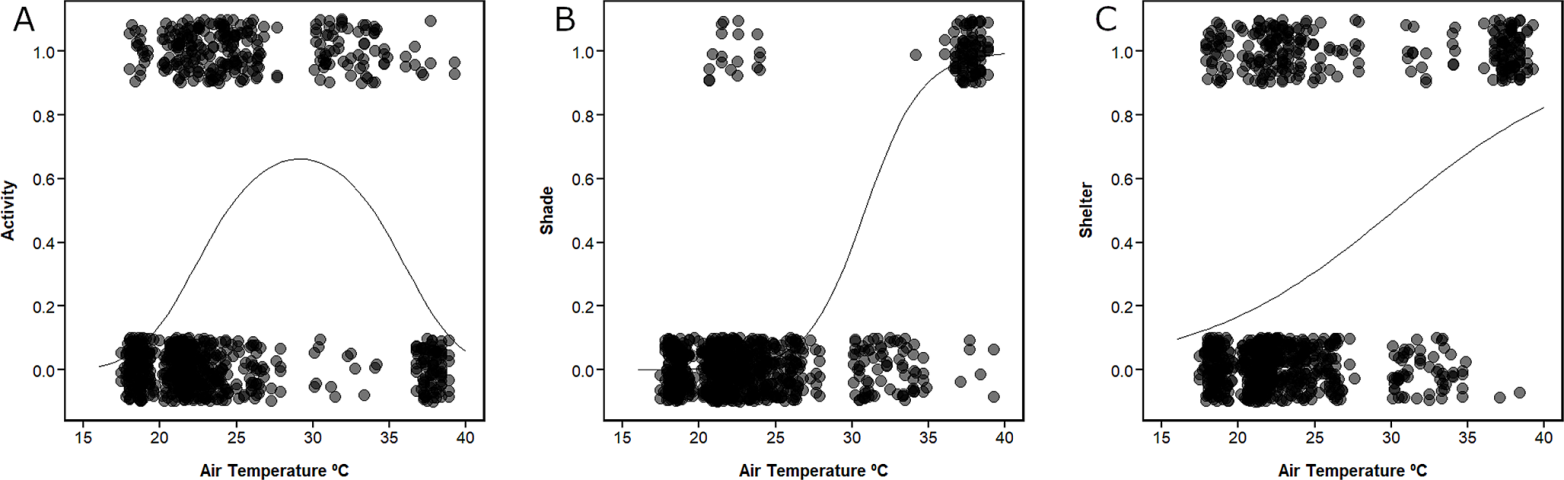
Butterfly **A** flight activity, **B** use of shade and **C** use of shelter against air temperature on the morning and afternoon of 19th July 2022 and five subsequent cooler days over a 15-day period at Pegsdon Hills and Hoo Bit nature reserve, Bedfordshire, UK. Points show data from individual butterflies, which can take values of 1 (observed flying, in the shade or < 5 m of shelter) or 0 (not flying, in the sun, or > 5 m from shelter). Points are plotted with jitter around the y-axis values for ease of interpretation. Solid lines show fitted logistic regressions, with the regression for shade **B** fitted using the mean value of shade coverage recorded during all surveys

Butterfly flight activity peaked at an air temperature of 30 °C (logistic regression, quadratic effect of air temperature, *n* = 926, *χ*^*2*^ = 139.63, *df* = 1, *p* < 0.001, Fig. 1A). The highest activity levels were recorded on the morning of 19th July 2022, where air temperatures ranged between 30.1 and 34.9 °C and 83% (64/77) of butterflies were flying (Supplementary data Fig. 3). However, activity levels dropped rapidly that afternoon, when temperatures ranged from 36.1 to 39.2 °C and only 10% (10/101) of butterflies were flying, compared to 28% (209/748) across all other lower temperature surveys (Fig. 1A).

### Question 2: does the probability of butterflies using shade increase at extreme high air temperatures?

At high temperatures, there was a significant increase in the number of butterflies found in the shade. At low air temperatures, even if large areas of shade were available, relatively low numbers of butterflies were found in shade (logistic regression, interaction between air temperature and area of shade, *n* = 926, *x*^*2*^ = 12.48, *df* = 1, *p* < 0.001, Fig. 1B). During the afternoon survey on 19th July 2022, 94% (95/101) of butterflies were found in the shade, compared to only 2% (20/825) in all other surveys (Supplementary data Fig. 3). During the hot afternoon survey, large multi-species groups of butterflies and other insects, including hoverflies, were observed sheltering together in the small available patches of shade. In one patch of shade in the scrub, more than 15 butterflies of at least nine species were recorded in an area less than 3 m^2^. Butterflies showed reduced activity levels in the shade, with only 7% (8/115) of butterflies observed flying, as opposed to 34% (275/811) of butterflies recorded in open areas.

### Question 3: does the probability of butterflies being found within 5 m of shelter increase at extreme high air temperatures?

Butterflies were more likely to be within 5 m of butterfly banks or scrub when air temperatures were higher (logistic regression, effect of air temperature, *n* = 926, *χ*^*2*^ = 162.40, *df* = 1, *p* < 0.001, Fig. 1C). During the high temperature afternoon, only 2% (2/101) of butterflies were recorded more than 5 m from scrub or butterfly banks (83 butterflies found near scrub, 16 found near butterfly banks), and both of these individuals were hidden within long grass (Supplementary data Fig. 1C). Butterflies on the banks were all resting on the shaded slopes, within or behind vegetation. This contrasts with all other surveys, when 77% (638/825) of butterflies were found in the open (Supplementary data Fig. 3).

### Question 4: does the difference between butterfly body temperature and air temperature reduce at extreme high air temperatures?

**Fig. 2:**
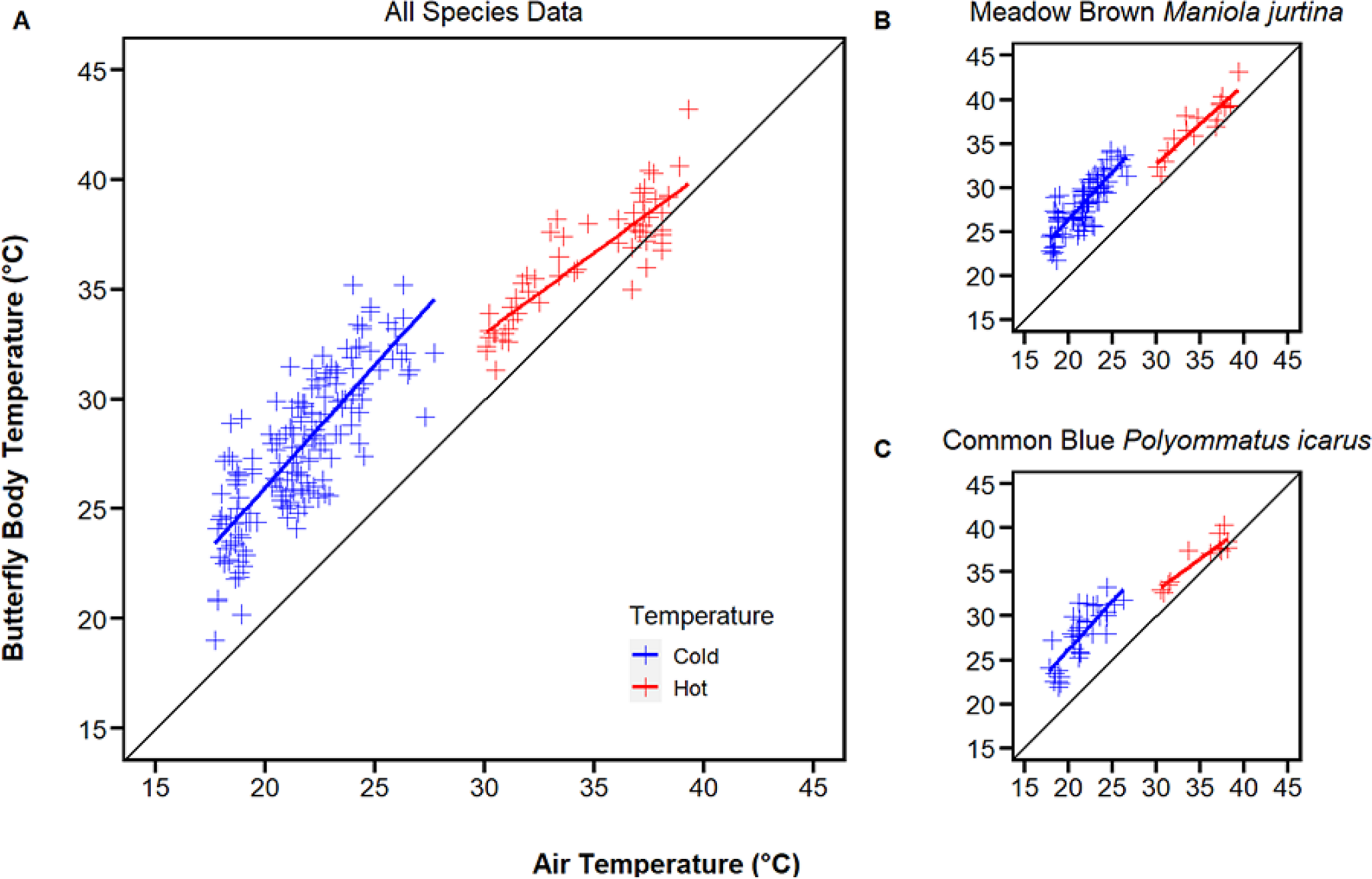
Response of butterfly body temperature to changes in air temperature recorded during the extreme high temperatures on 19th July 2022 (Hot, red) and five subsequent cooler days (Cold, blue) over a 15-day period on Pegsdon Hills and Hoo Bit nature reserve, Bedfordshire, UK for **A** all species and individually for **B** meadow brown *Maniola jurtina* and **C** common blue *Polyommatus icarus*, the two commonest species recorded during the survey

At the community level, the slope of the regression between body temperature and air temperature was shallower during the extreme high temperatures of 19th July 2022 than during the lower temperature surveys (linear regression, interaction between air temperature and survey day, *n* = 244, *F* = 16.81, *df* = 1, *p* < 0.001, Fig. 2A). The gradient of the regression on cooler days was 1.11, with butterfly body temperature increasing more quickly than the surrounding air temperature. On 19th July, the gradient of the regression was 0.73, with butterfly body temperature increasing more slowly than the surrounding air temperature. Data for both meadow brown (*Maniola jurtina*) and common blue (*Polyommatus icarus*) showed a similar pattern to the community-wide results, although neither showed a significant difference in slope between the high and low temperature surveys (meadow brown: *n* = 97, *F* = 1.30, *df* = 1, *p* = 0.257, Fig. 2B; common blue: *n* = 49, *F* = 3.17, *df* = 1, *p* = 0.082, Fig. 2C).

### Question 5: do butterflies select relatively cooler microclimate temperatures at extreme high air temperatures?

**Fig. 3:**
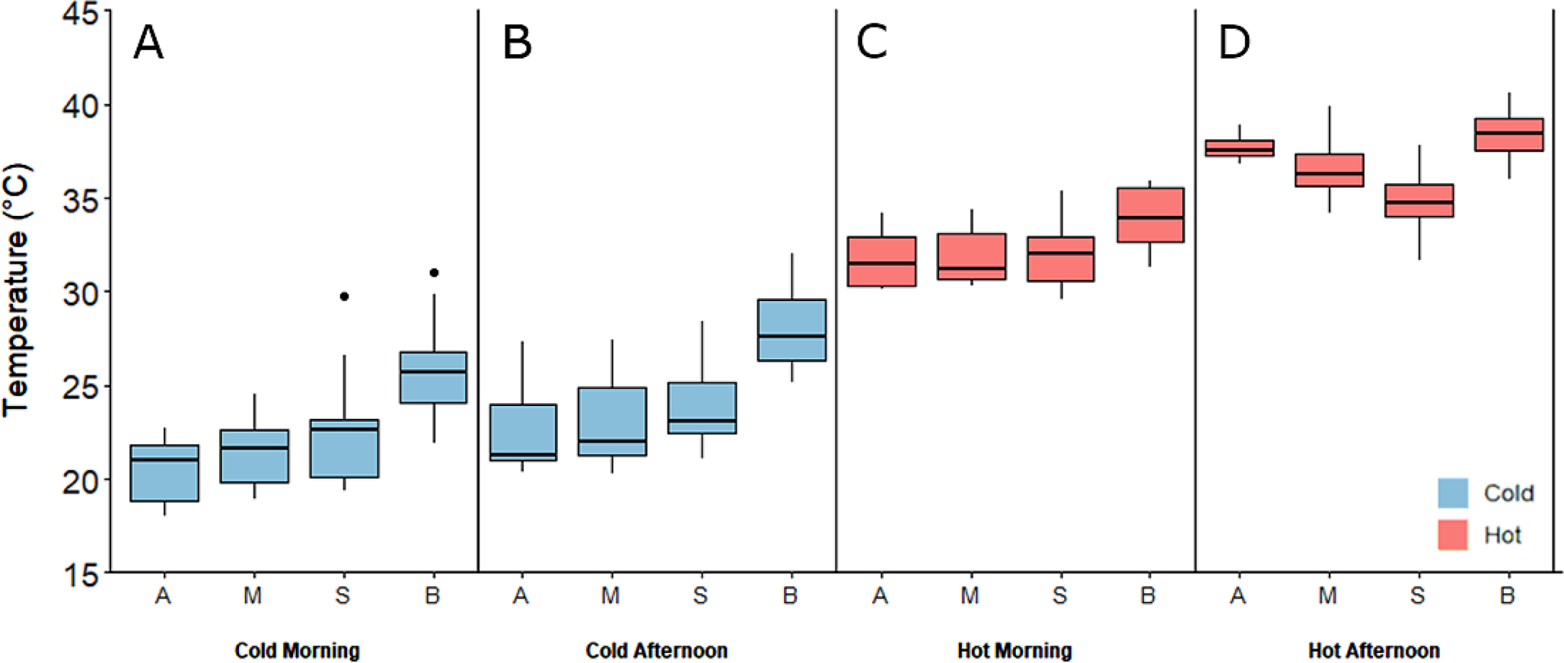
Variation in four different temperature measurements (**A** = air temperature, **M** = microclimate temperature, **S** = substrate temperature and **B** = butterfly body temperature) between surveys conducted during the extreme weather event on 19th July 2022 (Hot Morning and Hot Afternoon, red) and five subsequent cooler days (Cold Morning and Cold Afternoon, blue) over a 15-day period on Pegsdon Hills and Hoo Bit nature reserve, Bedfordshire, UK. Survey periods are arranged from left to right by increasing mean temperatures. Box and whisker plots show median values, with boxes representing the interquartile range and whiskers extending to the largest value no more than 1.5 × the interquartile range. Data outside of this range are plotted as points

A significant interaction was found between type of temperature measure (air temperature, microclimate temperature, substrate temperature, and butterfly body temperature) and survey time (Cold Morning, Cold Afternoon, Hot Morning and Hot Afternoon) on temperatures recorded during this study (two-way ANOVA, interaction between type of temperature measure and survey time, *n* = 280, *F* = 7.97, *df* = 9, 264, *p* < 0.001). During the ‘Cold Morning’, ‘Cold Afternoon’ and ‘Hot Morning’ surveys, the microclimate and substrate temperatures were similar to air temperature (Tukey post hoc tests, *p* > 0.05, Fig. 3A, B & C). However, in the ‘Hot Afternoon’ survey, substrate temperatures (Tukey post hoc test, *p* < 0.001) were significantly cooler than the surrounding air temperature (Fig. 3D, Supplementary data Table 2). As air temperature increased, the significant excess in body temperature compared to air temperature recorded in the ‘Cold Morning’ surveys (Tukey post hoc test, *p* < 0.001) reduced (Fig. 3A, B & C), to the point where the two temperature measures were not significantly different during the ‘Hot Afternoon’ survey (Tukey post hoc test, *p* = 0.997) (Fig. 3D, Supplementary data Table 2).

## Discussion

We found that during a recent extreme hot weather event in the UK, butterflies were less active and more likely to be found in the shade than on subsequent “normal” weather days. During the heatwave, butterflies made more use of shade provided by sheltering scrub and artificial butterfly banks than on other days. This study builds on a large existing body of literature, which demonstrates that butterflies and other invertebrates respond to high temperatures by seeking shade (Clench 1966; Kingsolver and Watt 1983; Kemp and Krockenberger 2004; Kleckova and Klecka 2016) and suggests that microclimate variability will be important to allow species to cope with future extreme heat events associated with climate change (Sunday et al. 2014; Woods et al. 2015; Kleckova and Klecka 2016; Sears et al. 2016). Here, for the first time, we observed the impact of temperatures above 40 °C in the UK on butterflies in the field, and the potential for management solutions to artificially create microclimate refugia.

Butterfly flight activity was highest on the morning of 19th July 2022, when air temperatures were high but not extreme (Supplementary data Fig. 3). Butterflies and other ectothermic organisms rely on the temperature of their environment to enable energetically demanding behaviour, such as flight (Shreeve 1984; Berwaerts and Van Dyck 2004; Merckx and Van Dyck 2005; Tsubaki and Samejima 2016; Hayes et al. 2019). Therefore, it is unsurprising that an initial increase in air temperature increased flight activity. By 08:40 on 19th July, air temperatures were already above 30 °C, higher than on any other survey day, and observations were more akin to a midday survey under ‘normal’ conditions. However, when temperatures rose above 35 °C during the afternoon, the majority of butterflies became inactive, and it appeared to be too hot for them to fly and carry out behaviours such as feeding, mating and defending territories (Fig. 1A) (Huey and Kingsolver 1989; Berwaerts and Van Dyck 2004; Sunday et al. 2014; Kearney et al. 2009; Woods et al. 2015; Evans et al. 2019; Harvey et al. 2020; Hill et al. 2021). Similarly, the open field within the survey area appeared to have become too hot for the insects (Supplementary data Fig. 1Ci), reducing counts of butterflies. Instead, during the afternoon survey of 19th July, the vast majority of butterflies were found in the shade, close to shelter, and were not flying (Supplementary data Fig. 3).

During surveys at lower temperatures, large patches of shade contained few, if any, butterflies. This demonstrates that species only made use of the shade when experiencing very high temperatures. The high densities of not just butterflies, but multiple species of insects, that we observed in the hottest period suggests that even small patches of shade may be vital for insect thermoregulation during extreme hot weather events (Kearney et al. 2009; Harvey et al. 2020; Thakur et al. 2020).

Our results comparing butterfly body and air temperature support the idea that butterflies were seeking shade and shelter to cool down during peak temperatures. Indeed, although different species of butterfly respond differently to changes in temperature (Angilletta 2009; Kleckova and Klecka 2016; Bladon et al. 2020), there appears to be a general overall change in the relationship between butterfly and air temperature at around 30 °C (Fig. 2). Below this, butterfly body temperatures warmed faster than the environment, whereas above this point they warmed slower than the environment, and the absolute difference between body temperature and air temperature was reduced. This different gradient in the relationship between butterfly body and air temperature below and above 30 °C (Fig. 2A) could result from a curved relationship, where butterflies gradually switch from seeking warm microclimates to seeking cool microclimates as air temperatures increase. Whether or not this change is more sudden or gradual, the inflection point is roughly in line with the shift in activity levels that we observed at around 30 °C, where butterflies became increasingly inactive and were less likely to fly (Fig. 1A). Furthermore, at the highest air temperatures, we recorded the only eight instances where butterfly body temperatures were below air temperature (Fig. 2A). This result suggests that conservation management which maximises the variety of habitats and microclimates that are available will continue to be important in the future, as the use of the environment for thermoregulation by butterflies changes with rising air temperatures.

The two species with the most data (meadow brown and common blue) showed a similar pattern to the community results (Fig. 2B & C), although this was not significant, likely due to the relatively small sample sizes available. It should be noted that analysing data together, from a whole community of butterflies, could mask intricacies of how individual species react to extreme temperatures (Angilletta 2009; Kleckova and Klecka 2016; Bladon et al. 2020). It is likely that observed trends are largely driven by the commonest species. However, all butterflies and moths recorded in this study are grassland species experiencing similar conditions and the community remained largely consistent across all surveys, with meadow brown being the most common species on every occasion (Supplementary data Table 1).

Further support for butterflies seeking shade and shelter to thermoregulate and cool down during hot conditions comes from the subset of butterflies where microclimate and substrate temperatures were also collected, alongside body and air temperatures. Under ‘normal’ weather conditions (Cold Morning and Cold Afternoon), individuals that were first observed perching during surveys were in microclimates and on substrates that were a similar temperature to ambient air temperatures, although substrate temperatures on the Cold Morning tended to be non-significantly warmer than air temperature (Supplementary data Table 2). This suggests that individual butterflies were, if anything, selecting warmer perch locations to heat up (Shreeve 1984; Kemp and Krockenberger 2004; Kleckova and Klecka 2016) and raise body temperatures above ambient levels (Fig. 3A & B). However, in the highest air temperatures on the afternoon of 19th July (Hot Afternoon), the substrates where we found butterflies were cooler than ambient air temperature (Supplementary data Table 2), a phenomenon we have not previously observed (Bladon et al. 2020, Laird-Hopkins & Ashe-Jepson et al. 2023, Toro-Delgado et al. 2024). This suggests butterflies were seeking microclimates with cooler conditions to lower their body temperatures (Clench 1966; Angilletta 2009; Kleckova and Klecka 2016) (Fig. 3D).

This study only collected data from one location and one extreme weather event. Despite this, our results demonstrate clear impacts of unusually high temperatures on a community of butterflies in the field. This provides valuable information which can be built on in future work during other extreme weather events, across multiple sites, delving further into the responses of specific species. Our results indicate that extreme temperatures, such as those experienced in the UK on 19th July 2022, are too warm for butterflies, and by implication other insects, to behave normally. Under these conditions they are forced to find shade and become inactive, unable to fly, feed or find mates. However, our results also demonstrate that actively managing for topography to produce shaded slopes and integrating patches of scrub within grassland could be a viable way to reduce the negative impacts of extreme weather events (Suggitt et al. 2012; Oliver 2014; Suggitt et al. 2015; Greenwood et al. 2016; Bladon et al. 2020). This could increase the time available to butterflies and other invertebrates for feeding and mate acquisition (Merckx and Van Dyck 2005; Kearney et al. 2009; Tsubaki and Samejima 2016; Hayes et al. 2019; Harvey et al. 2020), or at least reduce the risk of heat damage, and provide a refuge for insects to survive extreme heat (Suggitt et al. 2018; Thakur et al. 2020).

It is important to note that the observed changes in butterfly activity window may not be entirely negative. Although peak temperatures at midday limit activity levels, it could be that species can become active earlier in the morning and remain active longer into the evening. They may also benefit from an overall increase in activity level from the generally higher temperatures, and further study is needed to clarify whether reduced flight activity at peak temperatures is compensated for by increased activity at other times (Shreeve 1984; Kemp and Krockenberger 2004; Hill et al. 2021; Kral-O’Brien et al. 2021). This could explain why, in this study, the highest butterfly abundance and community richness was observed during the extreme temperature day of 19th July (Supplementary data Table 1). As individuals had ways to cool down and avoid the very highest temperatures, they may have benefitted overall from the heat wave, with butterfly numbers reducing later on, during the cooler days. Conversely, adverse effects of the extreme weather could have impacted butterfly fitness and caused the reduced numbers recorded on subsequent days (Thakur et al. 2022; Harvey et al. 2023). More research is needed to assess whether extreme weather events and bouts of inactivity have marked population effects, impacting reproductive success, feeding rates or mortality of affected individuals (Huey and Kingsolver 1989; Berwaerts and Van Dyck 2004; Sunday et al. 2014; Woods et al. 2015; Rohde et al. 2017; Evans et al. 2019; van Heerwaarden and Sgrò 2021; Harvey et al. 2023). We found that activity levels of butterflies increased again after the extreme temperatures of 19th July, suggesting individuals endured the high temperatures and to at least some extent recovered. Future bouts of extreme temperatures experienced over multiple, consecutive days, as expected under projected climate change (IPCC 2021), may well have more severe impacts (Harvey et al. 2023).

As climate change advances and regional temperatures rise (Newbold 2018; WWF 2020; IPCC 2021), our findings provide a stark warning about the potential impacts of extreme weather events on insects, and the need to plan and manage now for the future. High temperatures can cause large changes in activity levels, with possible impacts on population fitness, which are likely to become more severe when high temperature periods are longer in duration. However, varied topography and vegetation have the potential to provide microclimate refuges for affected species and managing for these at the reserve scale could buffer individuals against negative impacts. Whether through the construction of artificial earth banks or interspersing grassland with woody vegetation, providing shade and shelter adds to the repertoire of methods available to conservation practitioners to protect species from future projected climate change and extreme weather events.

## Electronic supplementary material

Below is the link to the electronic supplementary material.

Supplementary Material 1

## Data Availability

The data supporting the results have been archived in a public repository (Figshare) with the DOI and direct link 10.6084/m9.figshare.22083029 The code used for the analysis is available from the corresponding author on reasonable request.
