# Supplementary material for: Heatwave predicts a shady future for insects: impacts of an extreme weather event on a chalk grassland in Bedfordshire, UK
Source: J Insect Conserv. 2024 Feb 5;28(5):923–33. doi: 10.1007/s10841-024-00556-5 (PMC11489253; doi:10.1007/s10841-024-00556-5)
Supplement: Supplementary file 1 — Supplementary Material 1 [file 10841_2024_556_MOESM1_ESM.docx]

**Supplementary Material**
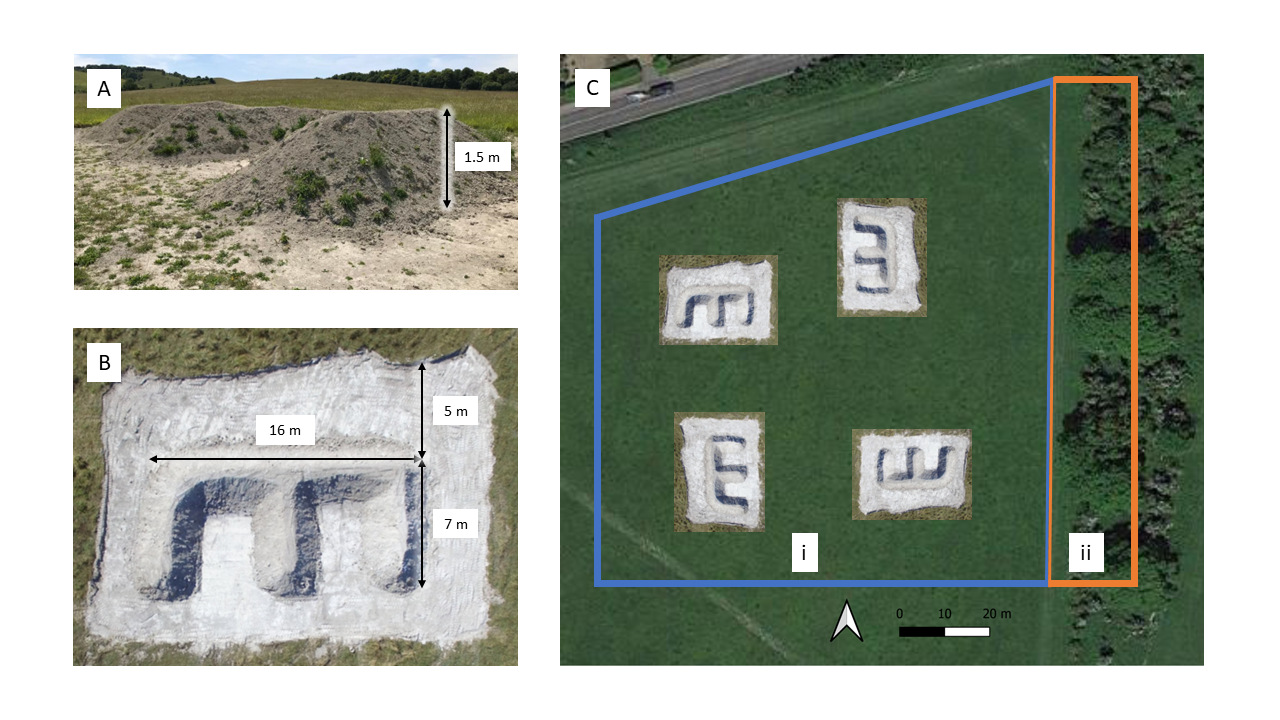


**Supplementary data Figure 1:** Butterfly banks at Pegsdon Hills and Hoo Bit nature reserve soon after they were built, showing **(A)** side on and **(B)** top-down orientations, as well as **(C)** an overview of the project survey area. The blue box delineates **(Ci)** an open grassland field containing the four butterfly banks. The orange box delineates **(Cii)** a scrub patch on the eastern edge of the field. All delineated areas represent the total survey area for this study.

**
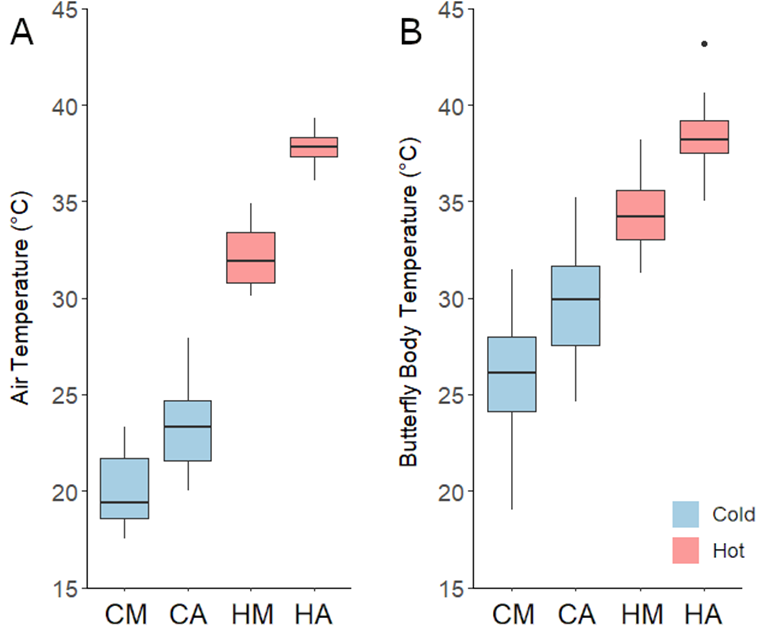
**

**Supplementary data Figure 2:** The **(A)** air temperature and **(B)** butterfly body temperature recorded on the morning and afternoon of 19^th^ July 2022 (HM = Hot Morning & HA = Hot Afternoon, Red) and five subsequent cooler days (CM = Cold Morning & CA = Cold Afternoon, Blue) over a 15-day period at Pegsdon Hills and Hoo Bit nature reserve, Bedfordshire, UK. Box and whisker plots show median values, with boxes representing the interquartile range and whiskers extending to the largest value no more than 1.5 × the interquartile range. Data outside of this range are plotted as outlying points.

**
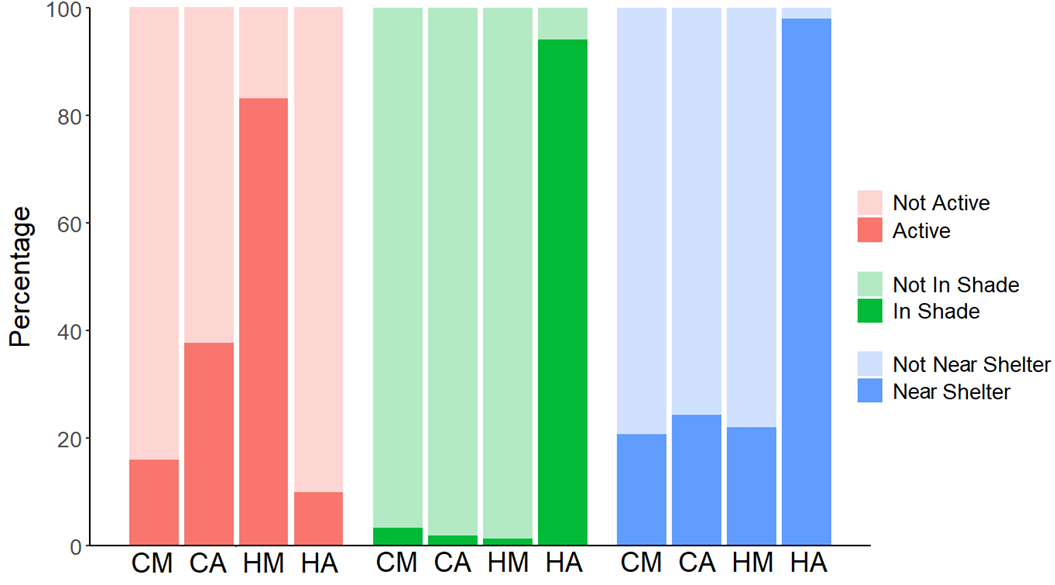
**

**Supplementary data Figure 3:** The percentage of butterflies flying (active) when first observed (red), located in the shade (green), and located within 5 m of a sheltering butterfly bank or scrub (blue), during surveys carried out on the morning and afternoon of 19^th^ July 2022 (HM = Hot Morning & HA = Hot Afternoon) and five subsequent cooler days (CM = Cold Morning & CA = Cold Afternoon) over a 15-day period at Pegsdon Hills and Hoo Bit nature reserve, Bedfordshire, UK.

| Species | July  19th  am | July  19th  pm | July  22nd  am | July  22nd  pm | July  25th  am | July  25th  pm | July  30th  am | July  30th  pm | August  1st  am | August  1st  pm | August  3rd  am | August  3rd  pm | **Overall Total** |
| --- | --- | --- | --- | --- | --- | --- | --- | --- | --- | --- | --- | --- | --- |
| 6-spot burnet moth (*Zygaena filipendulae*) | 7 | 1 | 0 | 1 | 0 | 0 | 0 | 0 | 0 | 0 | 0 | 0 | 9 |
| Brimstone (*Gonepteryx rhamni*) | 0 | 1 | 0 | 0 | 0 | 0 | 0 | 0 | 0 | 0 | 0 | 0 | 1 |
| Brown argus (*Aricia agestis*) | 0 | 0 | 0 | 0 | 0 | 0 | 0 | 1 | 0 | 0 | 0 | 0 | 1 |
| Chalkhill blue (*Polyommatus coridon*) | 5 | 6 | 7 | 13 | 15 | 3 | 2 | 5 | 2 | 6 | 7 | 4 | 75 |
| Clouded yellow (*Colias croceus*) | 0 | 0 | 0 | 0 | 0 | 0 | 1 | 0 | 0 | 0 | 0 | 0 | 1 |
| Common blue (*Polyommatus icarus*) | 13 | 24 | 7 | 13 | 7 | 10 | 13 | 15 | 3 | 10 | 5 | 3 | 123 |
| Dark green fritillary (*Speyeria aglaja*) | 1 | 0 | 0 | 0 | 0 | 0 | 0 | 0 | 0 | 0 | 0 | 0 | 1 |
| Gatekeeper (*Pyronia Tithonus*) | 4 | 7 | 7 | 17 | 6 | 22 | 5 | 5 | 2 | 6 | 5 | 6 | 92 |
| Large white (*Pieris brassicae*) | 1 | 0 | 0 | 1 | 0 | 1 | 0 | 1 | 0 | 1 | 0 | 0 | 5 |
| Marbled white (*Melanargia galathea*) | 4 | 6 | 0 | 2 | 1 | 0 | 3 | 2 | 0 | 0 | 0 | 0 | 18 |
| Meadow brown (*Maniola jurtina*) | 30 | 32 | 33 | 43 | 42 | 42 | 44 | 51 | 56 | 54 | 57 | 54 | 538 |
| Peacock (*Aglais io*) | 3 | 0 | 0 | 0 | 0 | 0 | 0 | 0 | 0 | 0 | 0 | 0 | 3 |
| Small heath (*Coenonympha pamphilus*) | 0 | 1 | 1 | 0 | 0 | 0 | 1 | 0 | 0 | 0 | 0 | 0 | 3 |
| Small or Essex skipper (*Thymelicus sylvestris/lineola*) | 9 | 20 | 2 | 2 | 1 | 2 | 0 | 0 | 0 | 0 | 0 | 2 | 38 |
| Small tortoiseshell (*Aglais urticae*) | 0 | 0 | 0 | 0 | 0 | 0 | 2 | 2 | 0 | 1 | 0 | 1 | 6 |
| Small white (*Pieris rapae*) | 0 | 3 | 0 | 2 | 0 | 0 | 0 | 1 | 0 | 3 | 0 | 3 | 12 |
| **Total abundance** | **77** | **101** | **57** | **94** | **72** | **80** | **71** | **83** | **63** | **81** | **74** | **73** | **926** |

**Supplementary data Table 1:** The number of butterflies and day-flying moths recorded during this study. Numbers of each species are given for each individual morning (am) and afternoon (pm) survey completed over a 15-day period at Pegsdon Hills and Hoo Bit nature reserve, Bedfordshire, UK. Essex and Small Skipper butterflies were recorded together as they need to be caught and have their antennae examined in order to accurately distinguish between the two species.

**Supplementary data Table 2:** p values for Tukey post hoc tests, comparing air temperatures recorded during surveys on the morning and afternoon of July 19^th^ 2022 (Hot Morning & Hot Afternoon) and five subsequent cooler days (Cold Morning & Cold Afternoon) with microclimate temperatures, substrate temperatures and butterfly body temperatures recorded during the same surveys, completed over a 15-day period at Pegsdon Hills and Hoo Bit nature reserve, Bedfordshire, UK. * indicates a significant p value, <0.05.

| **Air Temperature** | **Microclimate Temperature** | **Substrate Temperature** | **Body Temperature** |
| --- | --- | --- | --- |
| Cold Morning | 0.948 | 0.061 | < 0.001 * |
| Cold Afternoon | 0.999 | 0.887 | < 0.001 * |
| Hot Morning | 0.999 | 0.999 | 0.274 |
| Hot Afternoon | 0.644 | < 0.001 * | 0.997 |
